# Supplementary material for: Sub-pangenome analysis reveals structural variants associated with fruit color and bacterial wilt resistance in eggplant
Source: Nat Commun. 2026 Feb 23;17:3075. doi: 10.1038/s41467-026-69764-8 (PMC13039718; doi:10.1038/s41467-026-69764-8)
Supplement: Supplementary file 1 — Supplementary Information [file 41467_2026_69764_MOESM1_ESM.pdf]

Sub-pangenome analysis reveals structural variants associated with fruit color and bacterial wilt resistance in eggplant

You *et al.*

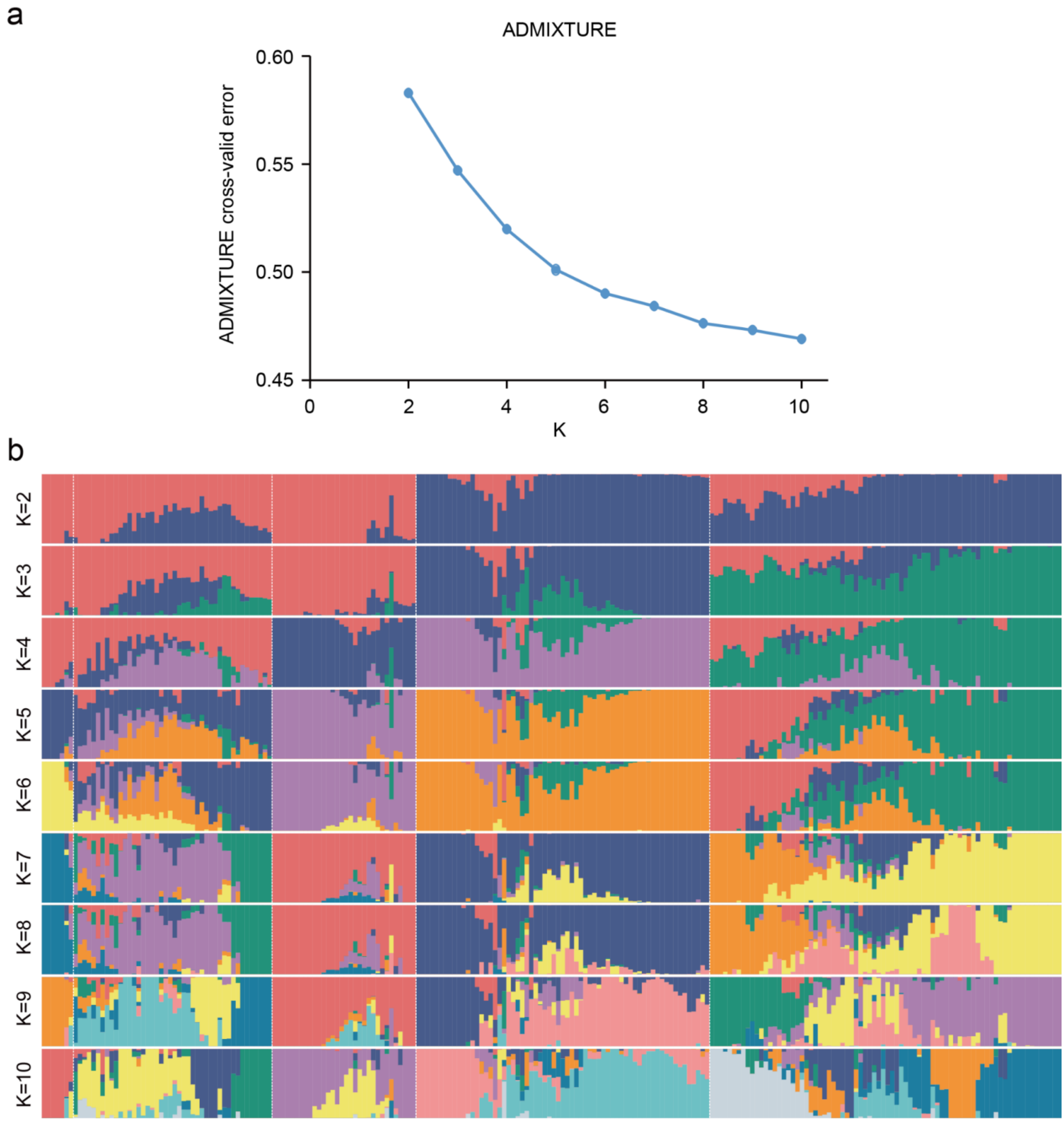

Supplementary Fig. 1. Admixture analysis of the population structure of eggplant. a, Cross-valid error of ADMIXTURE from K=2-10. **b**, Ancestral component analysis of eggplant accessions with ADMIXTURE for K=2-10. Source data are provided as a Source Data file.

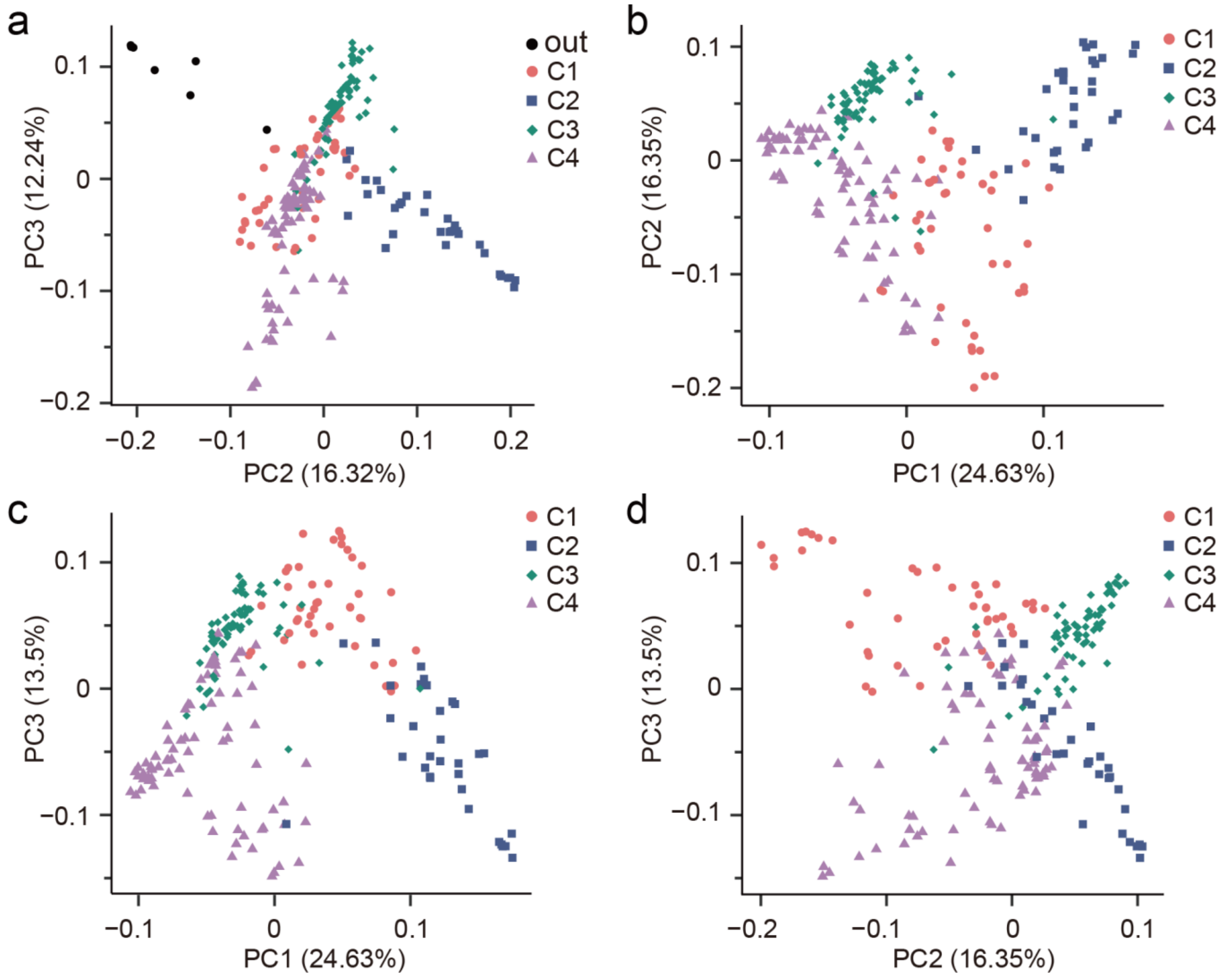

Supplementary Fig. 2. Principal component analysis (PCA) of eggplant population structure. a, PC2 *vs.* PC3 (including outgroup). **b**, PC1 *vs.* PC2 (excluding outgroup). **c**, PC1 *vs.* PC3 (excluding outgroup). **d**, PC2 *vs.* PC3 (excluding outgroup). The first three principal components are plotted. Groups identified with the phylogenetic tree and ADMIXTURE analysis are represented in different colored shapes. Source data are provided as a Source Data file.

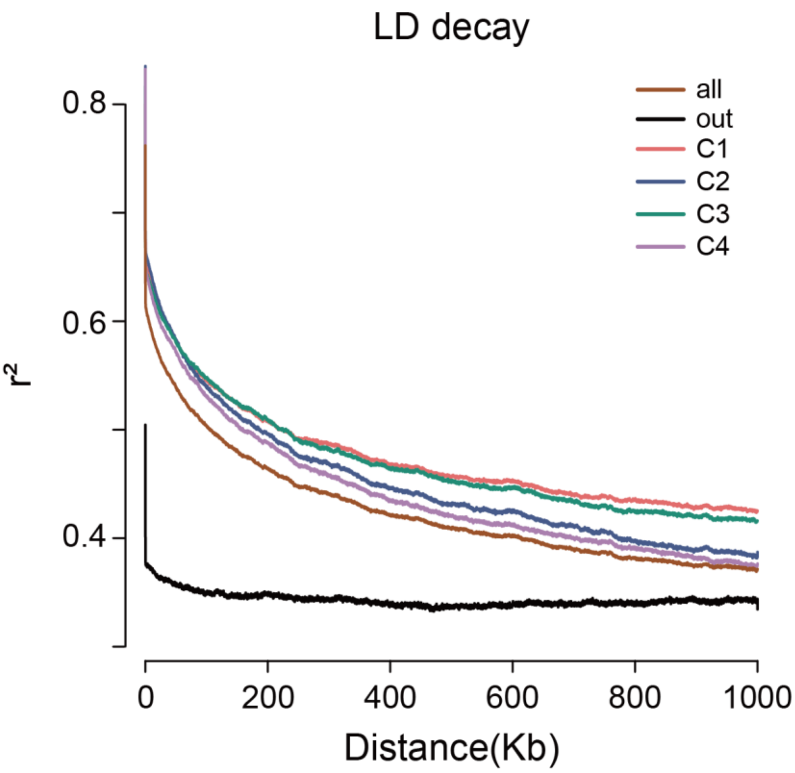

Supplementary Fig. 3. Linkage disequilibrium (LD) decay analysis in eggplant subpopulations. Linkage disequilibrium (LD) decay was calculated using PopLDdecay based on 153,934 high-quality SNPs. The colors represent subpopulations identified with the phylogenetic tree and ADMIXTURE analysis.

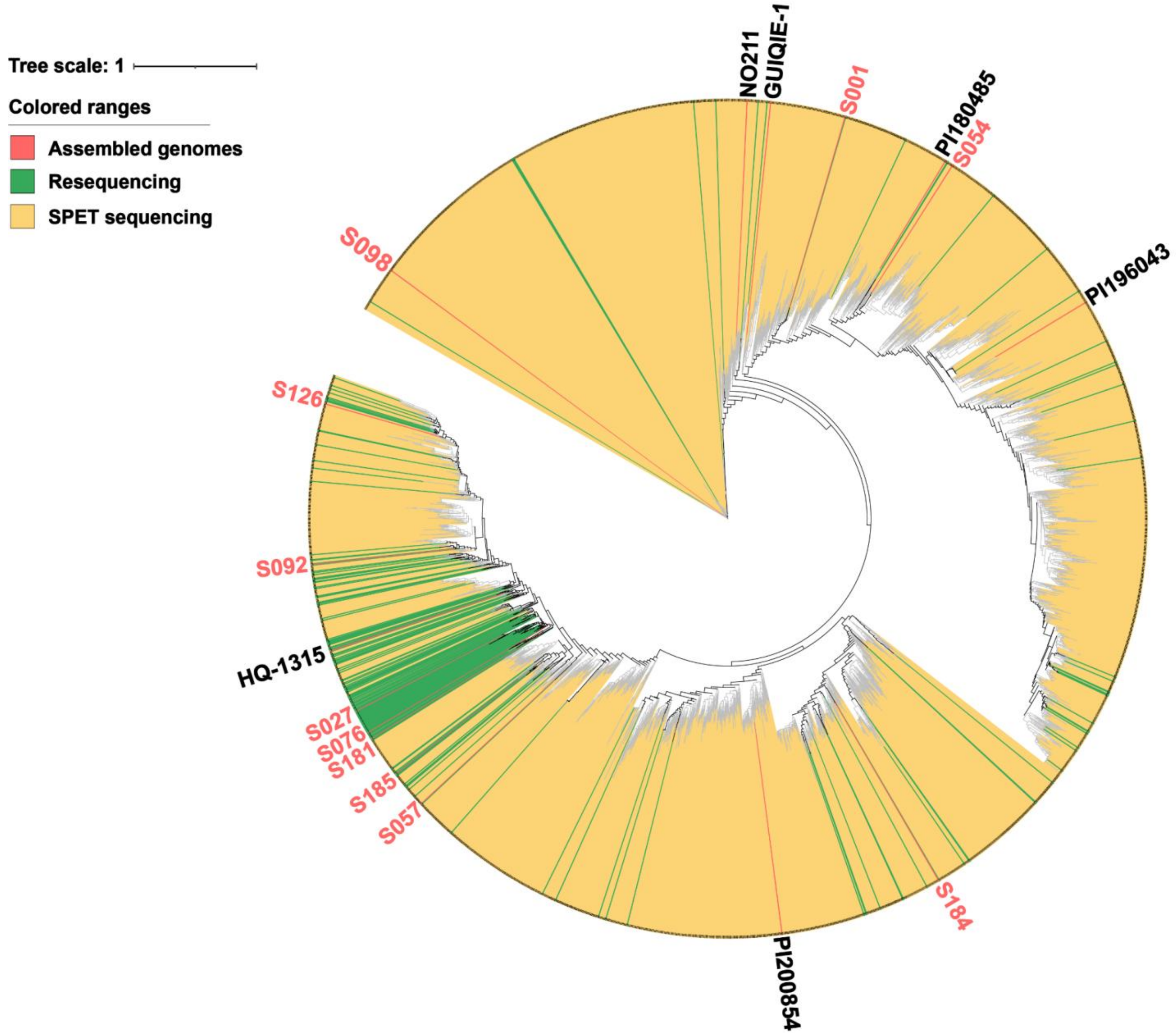

Supplementary Fig. 4. A phylogenetic tree covering 3,673 global eggplant accessions. A total of 710 SNPs were used to construct a maximum likelihood tree with IQ-tree (v1.6.12) under the best-fit substitution model selected by ModelFinder. The light yellow color (background) indicates samples with published sequencing data obtained through single primer enrichment technology (SPET). Samples with resequencing data are highlighted in green; the newly assembled genomes are highlighted in red; the published genomes are highlighted in black.

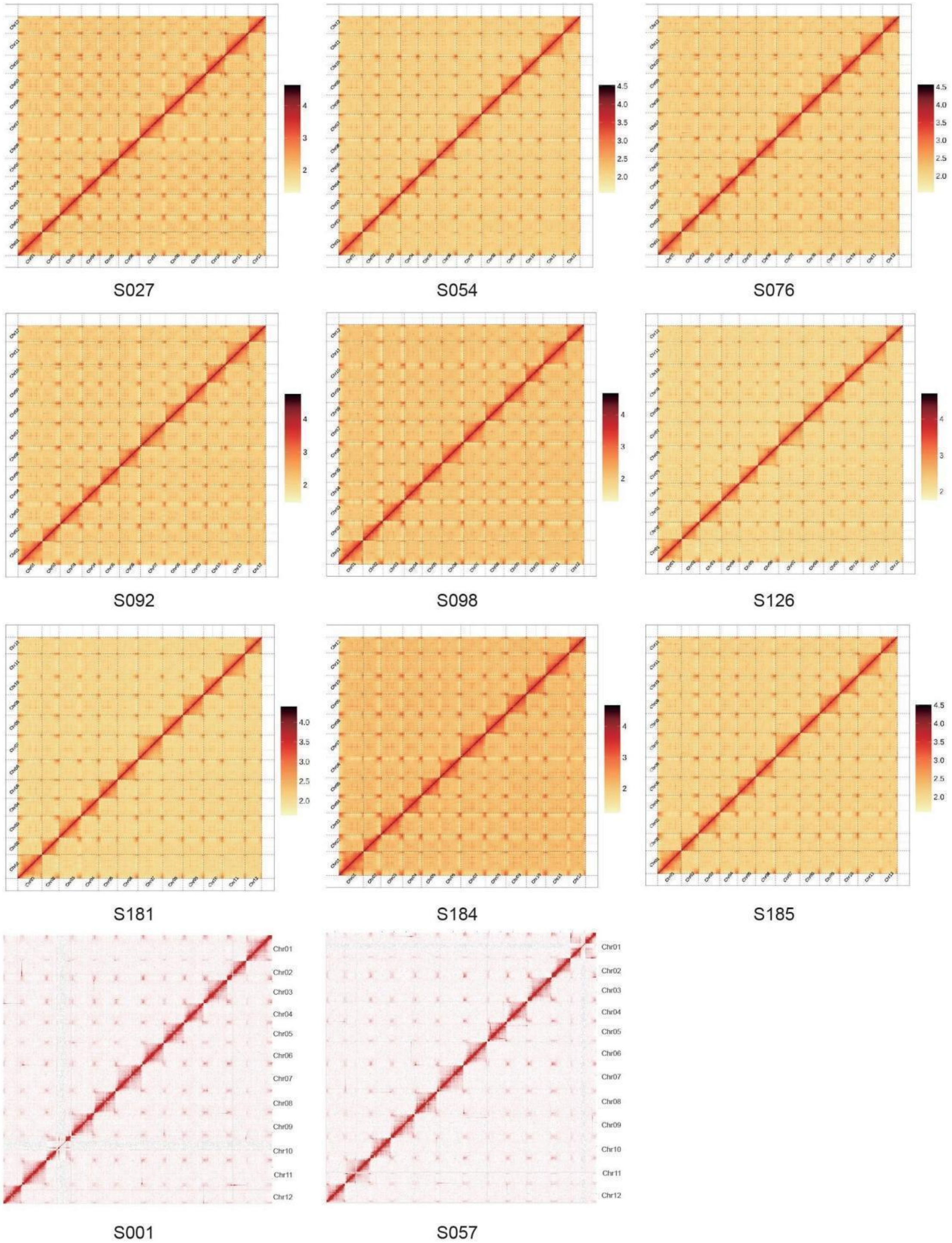

Supplementary Fig. 5. Hi-C maps of the 11 sequenced eggplant accessions.

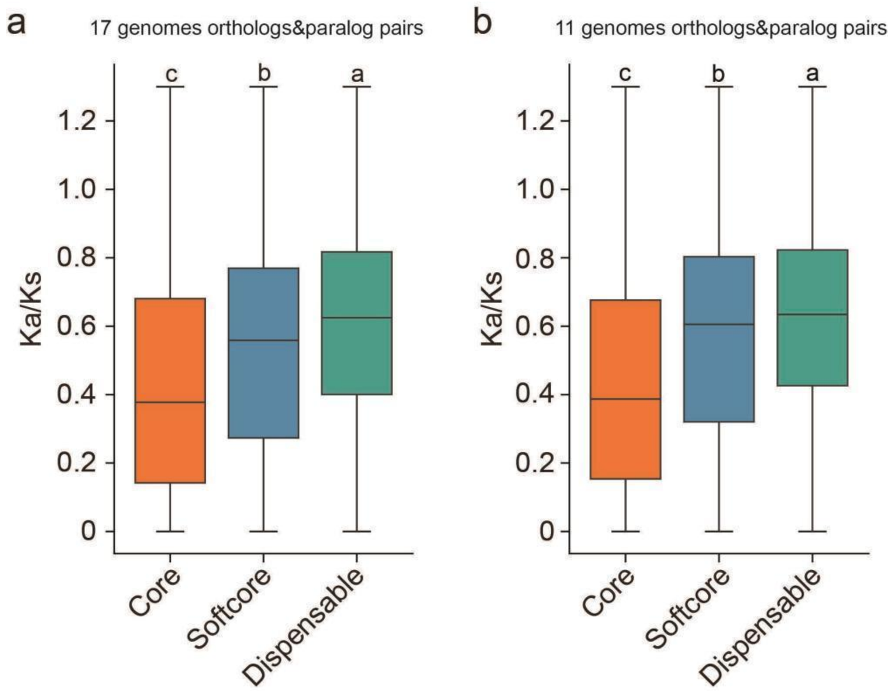

Supplementary Fig. 6. Comparison of K_a/K_s values among core, softcore, and dispensable genes. **a**, Comparison of K_a/K_s values among core (n=911,278), softcore (n=188,021), and dispensable genes (n=271,168) considering both ortholog and paralog pairs for the pangenome version of 17 genomes. **b**, Comparison of K_a/K_s values among core (n=439,403), softcore (n=52,374), and dispensable (n=63,993) genes considering both ortholog and paralog pairs for the pangenome version of 11 genomes. In boxplots (**a, b**), the lower and upper edges of the box represent the first and third quartiles, respectively, and the central line indicates the median. The whiskers extend to the smallest and largest values within 1.5 × interquartiles range (IQR). Different letters indicate significant differences (Tamhane's T2 test, two sided, $p < 0.05$). Source data are provided as a Source Data file.

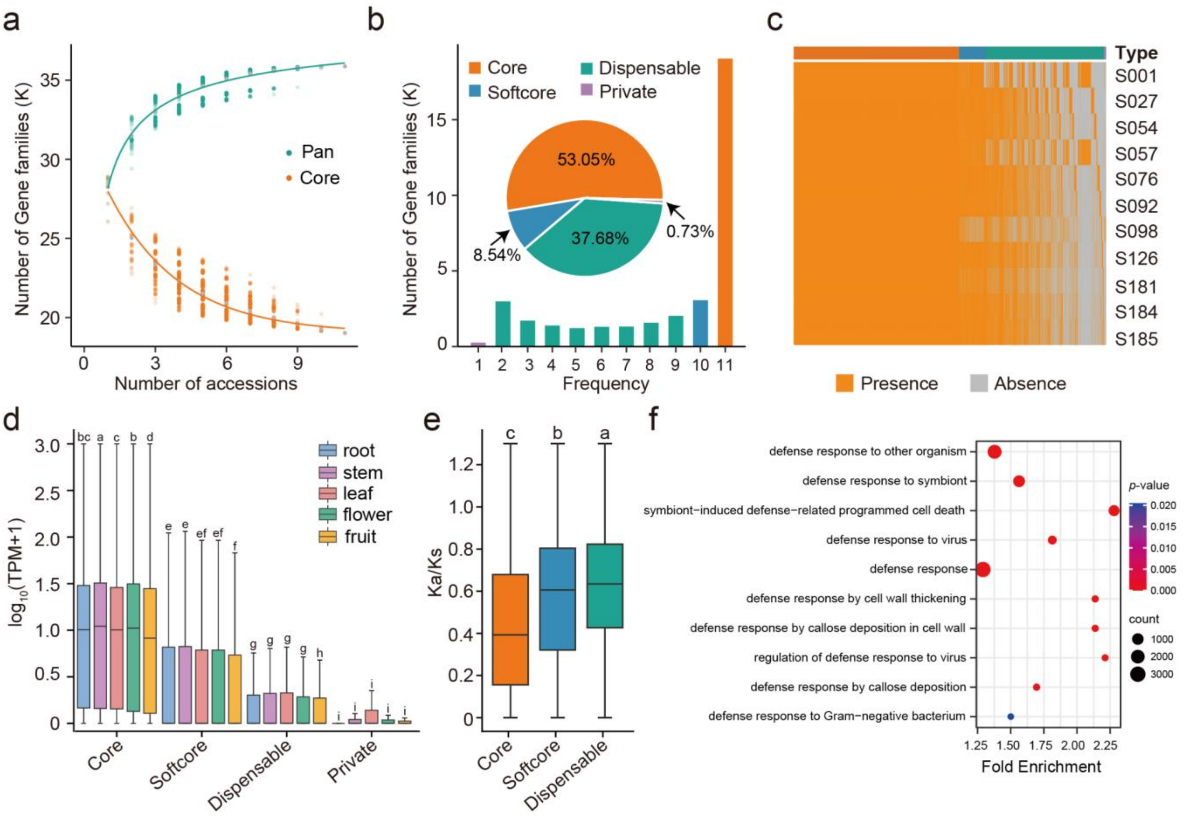

Supplementary Fig. 7. Pangenome analysis of 11 *de novo* sequenced eggplant accessions. a, Number of pan and core gene families considering 1-11 genomes. **b**, Frequencies and proportions of core, softcore, dispensable, and private gene families. **c**, Compositions of each of the 11 genomes. Colors are explained in b. Every row represents an accession. **d**, Comparison of gene expressions in five organs among core, softcore, dispensable, and private genes. Gene expressions were calculated for the 11 *de novo* genomes due to availability of transcriptome data. **e**, Comparison of K_a/K_s values among core (n=435,148), softcore (n=52,113), and dispensable (n=63,586) genes. In boxplots (**d, e**), the lower and upper edges of the box represent the first and third quartiles, respectively, and the central line indicates the median. The whiskers extend to the smallest and largest values within 1.5 × IQR. Different letters indicate significant differences (Tamhane's T2 test, two sided, $p < 0.05$). **f**, Gene Ontology (GO) terms significantly enriched in dispensable gene families and associated with defense. The GO terms for dispensable families were compared against the background of all GO terms associated with all genes of the 11 genomes. Circle sizes indicate different GO counts (number of genes associated with a GO in the dispensable genes); fold enrichment score was calculated as the percentage of a GO in dispensable genes relative to that in all genes; circle colors indicate different p values in -$\log_{10}$ scale (hypergeometric test, one sided). Source data are provided as a Source Data file.

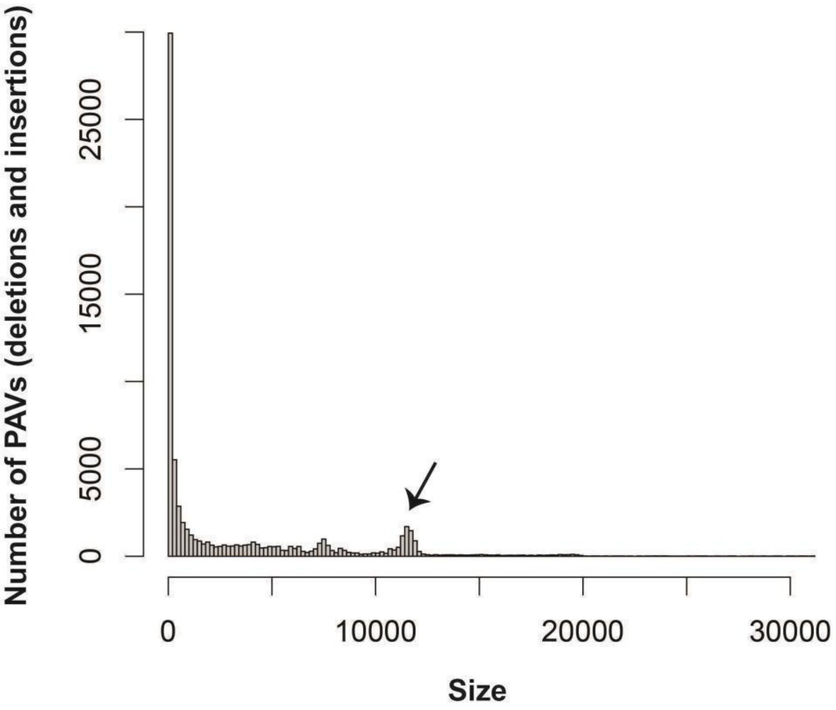

Supplementary Fig. 8. Length distribution of the PAVs (deletions and insertions). The arrow indicates a peak around 12 kb likely corresponding to the length of LTR (long terminal repeat) retrotransposons.

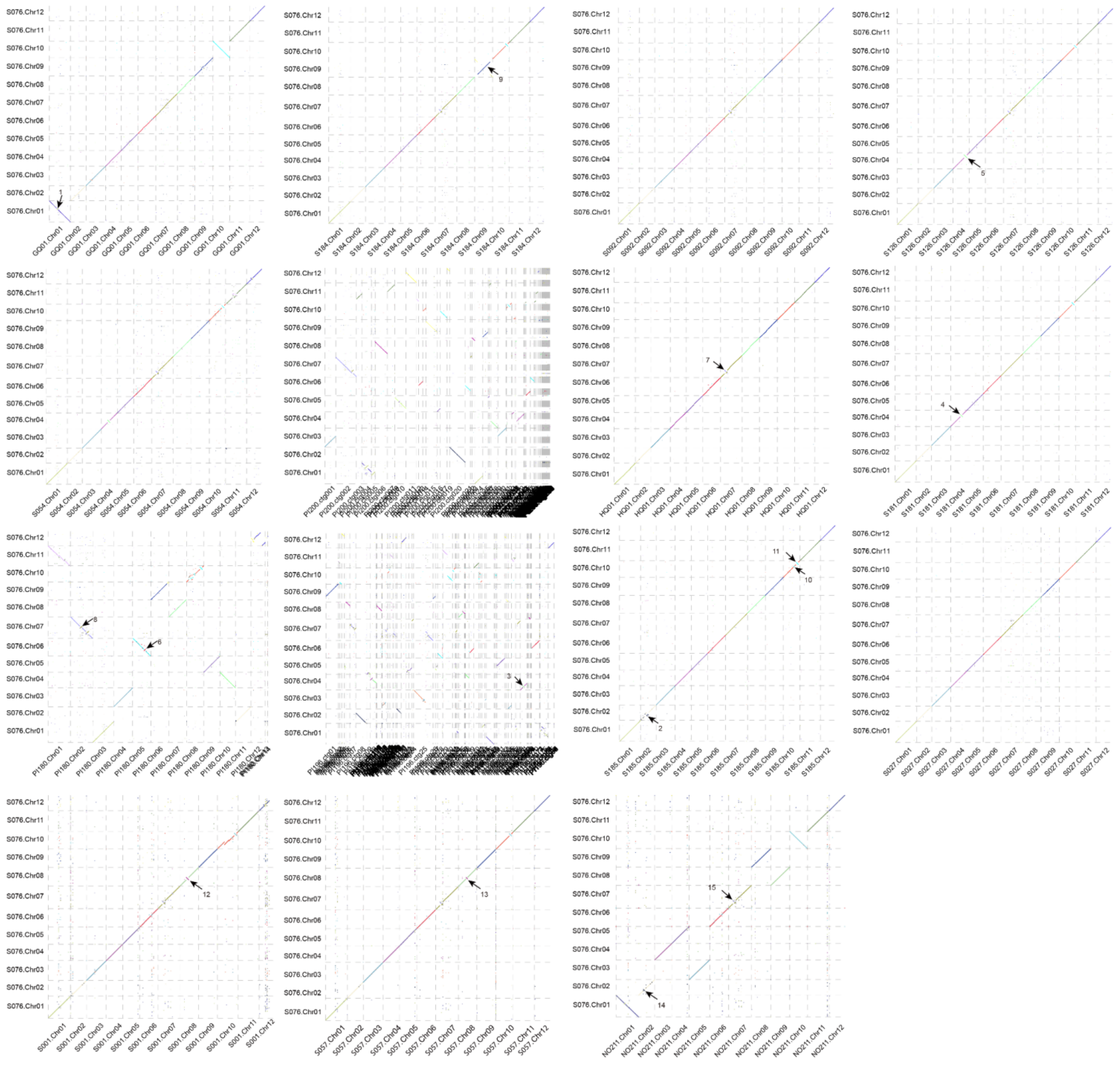

Supplementary Fig. 9. Genome synteny maps between the reference genome 'S076' and each of the 15 eggplant genomes. A total of 15 large inversions (approximately 5 Mb or larger), highlighted by arrows, which are segregating within the eggplant population.

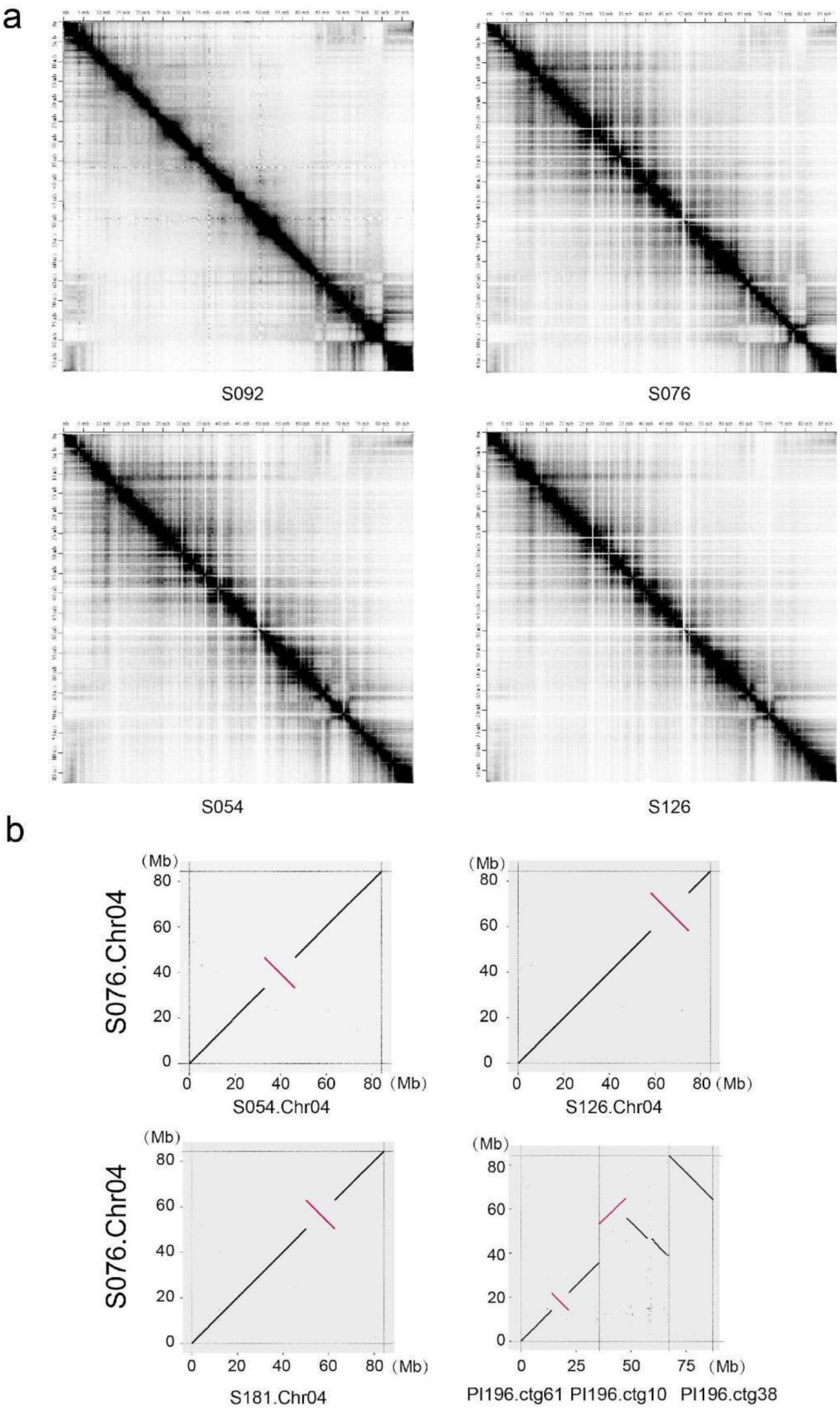

Supplementary Fig. 10. Examples of using Hi-C maps and synteny maps to verify large inversions. a, Hi-C maps for chromosome 10 from four eggplant accessions. A notable 12.4 Mb inversion distinctly alters the Hi-C contact patterns between accessions with the inversion (S092 and S076) and those without it (S054 and S126). **b**, Pairwise synteny maps of chromosome 4 displays four large inversions.

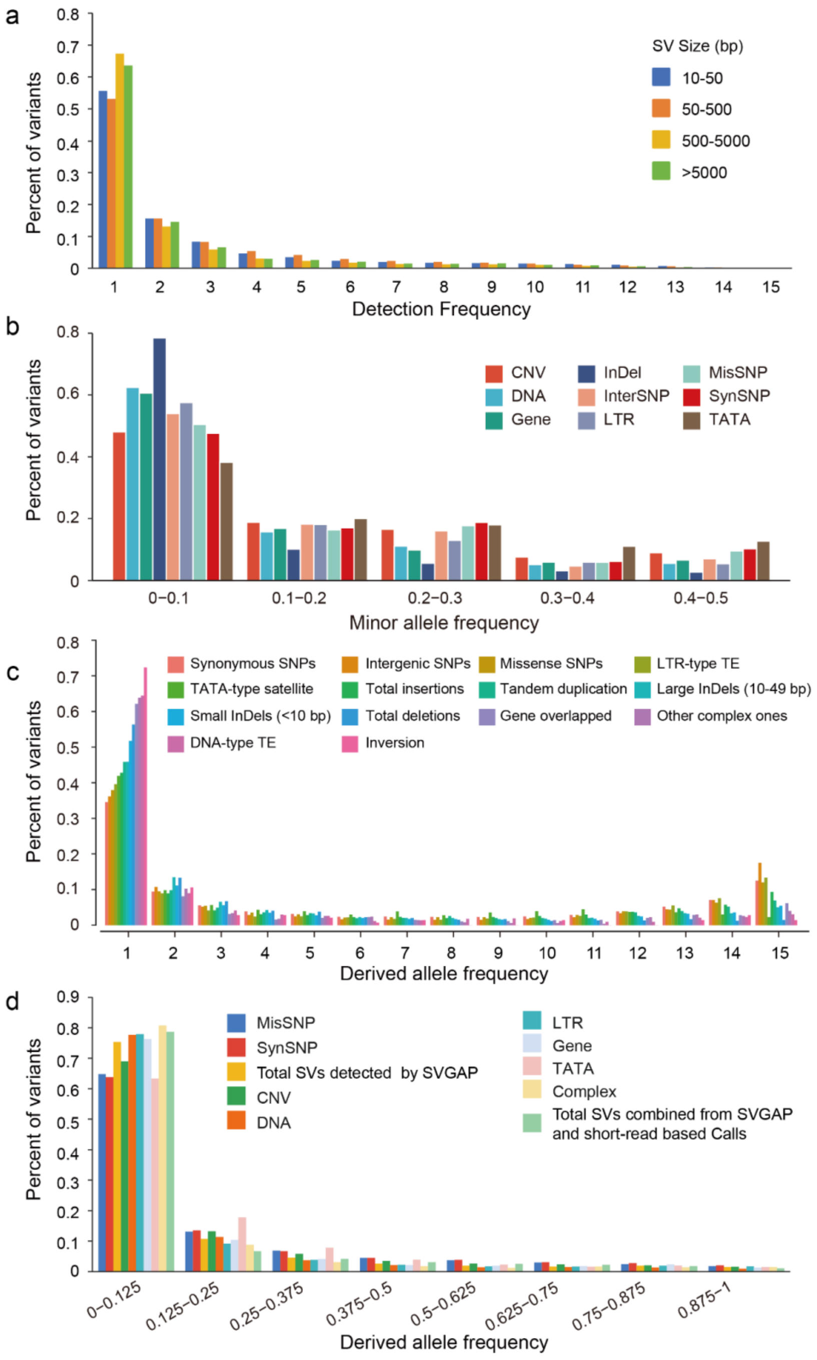

Supplementary Fig. 11. Population characteristics and fitness analysis of SVs in eggplant. a, The detection frequency of PAVs (deletions and insertions) across 15 eggplant genome assemblies (with S076 as the reference). Approximately 81.5% of variants are only detected in one or two assemblies. **b**, Minor allele frequency of different SV types compared to presumably neutral sSNPs and nSNPs in 15 eggplant genomes. **c**, The derived allele frequency for different kinds of genomic variations. *Solanum violaceum* was used as the outgroup to infer the ancestral state. **d**, Derived allele frequency for different types of SVs in 219 eggplant accessions.

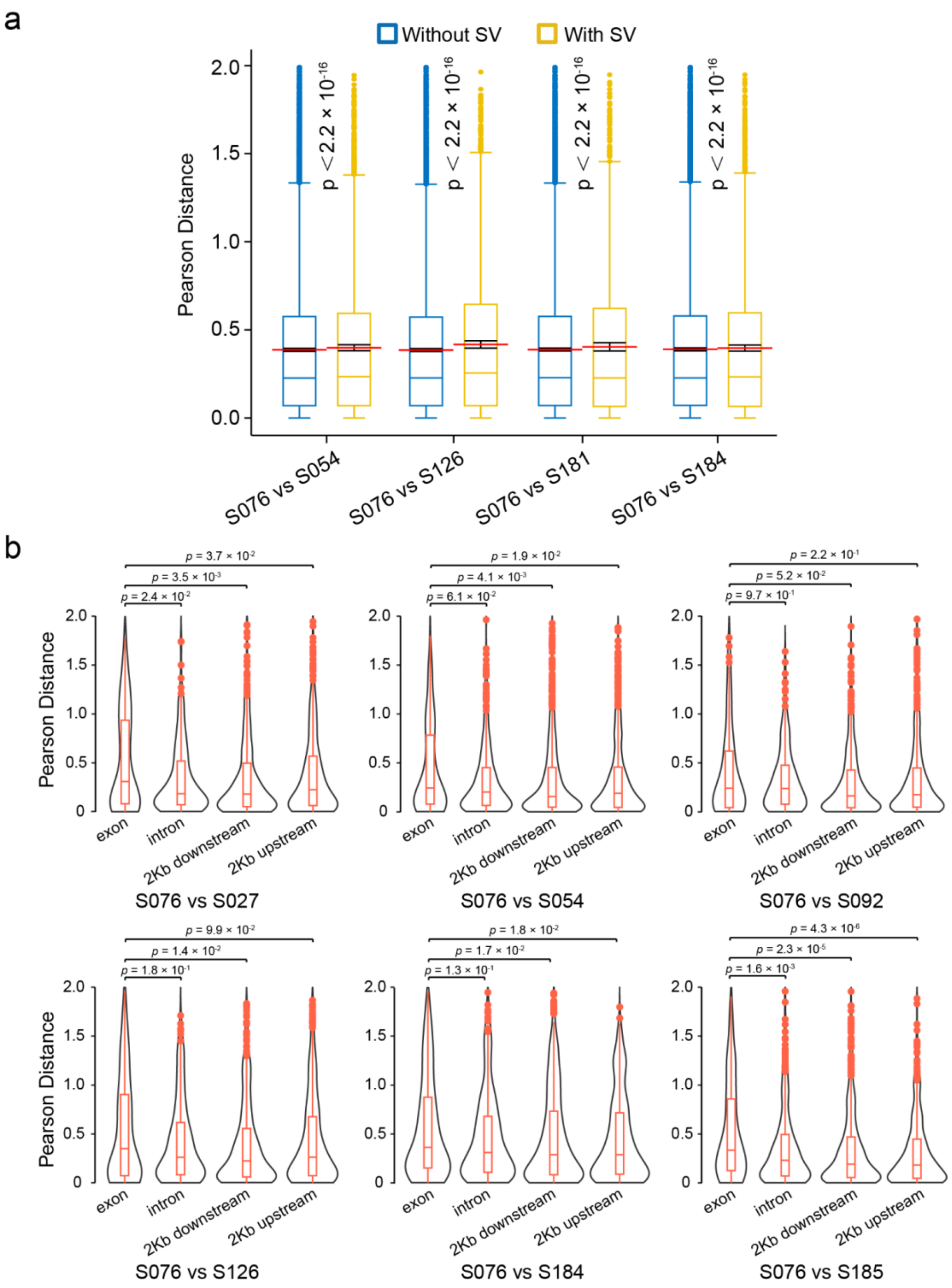

Supplementary Fig. 12. Expression variation measured by Pearson distance between gene sets with or without SVs. a, Expression variation measured by Pearson distance between two gene sets: one with at least one SV within 2 kb and one without. **b**, Expression variation measured by Pearson distance between two gene sets with SVs overlapping different genomic features. In boxplots (**a, b**), the lower and upper edges of the box represent the first and third quartiles, respectively, and the central line indicates the median. The whiskers extend to the smallest and largest values within 1.5 × IQR. Significant levels were determined using a two-sided Wilcoxon test.

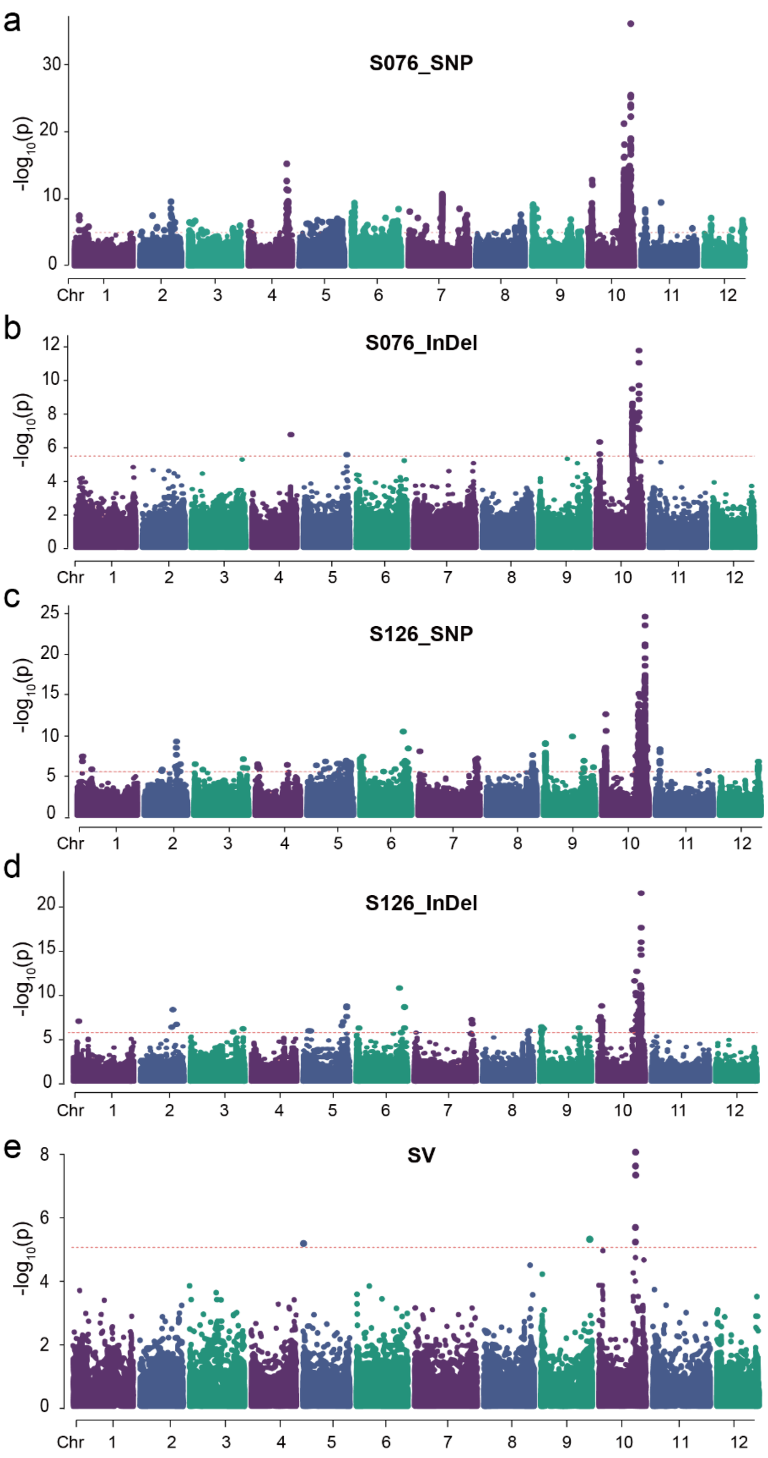

Supplementary Fig. 13. GWAS analysis for fruit color in eggplant. a, GWAS based on SNPs using S076 as the reference. **b**, GWAS based on InDels using S076 as the reference. **c**, GWAS based on SNPs using S126 as the reference. **d**, GWAS based on InDels using S126 as the reference. **e**, GWAS based on a unique SV set using S076 as the reference. All analyses reveal a significant signal on chromosome 10. *P* values were calculated using a two-sided Wald test based on a linear mixed model.

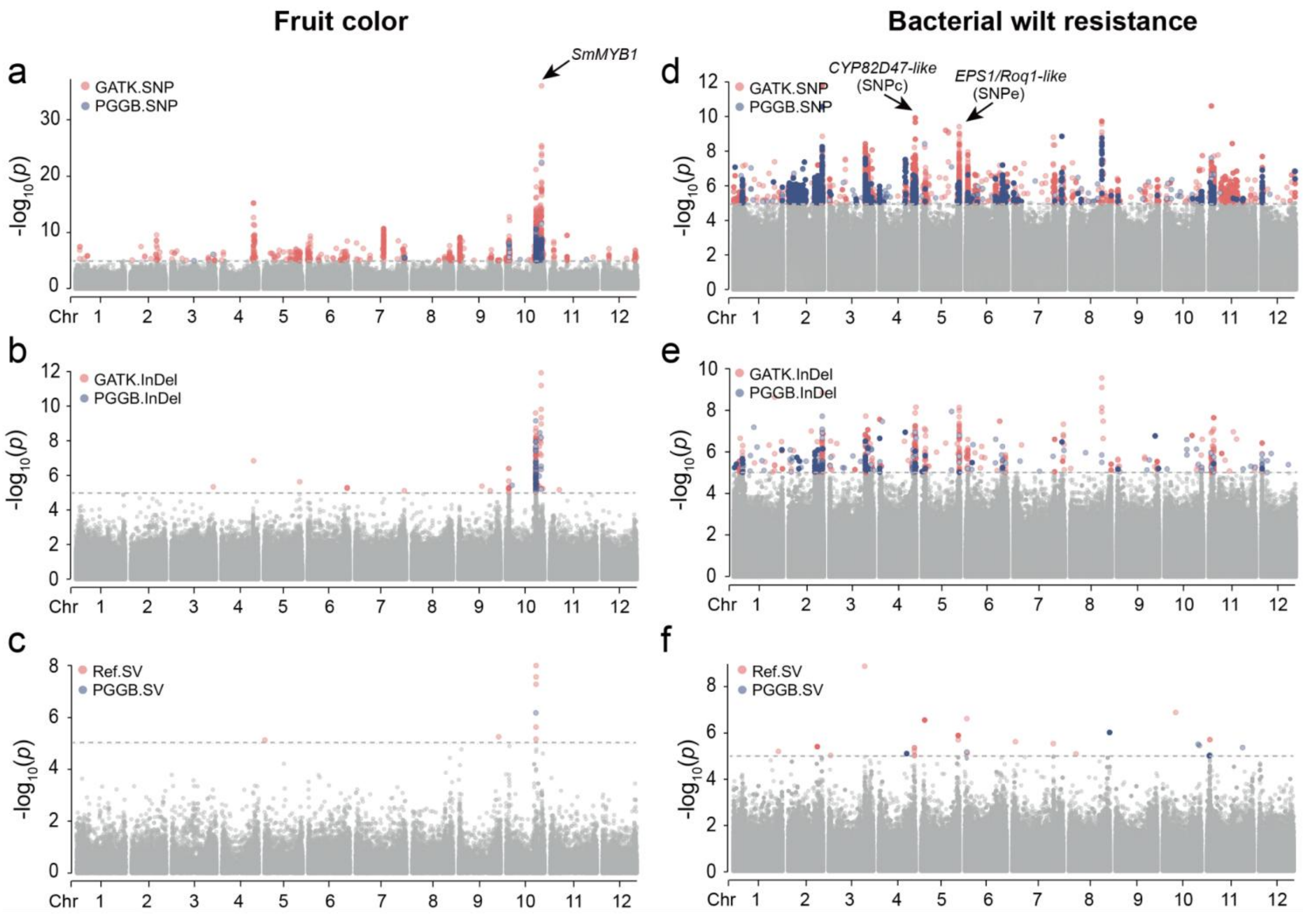

Supplementary Fig. 14. GWAS analysis for fruit color and bacterial wilt resistance in eggplant with reference based (GATK, SVGAP and Panpop) and non-reference based (PGGB) methods. **a**, **b**, **c**, Genome-wide association analyses based on SNPs, InDels, and SVs for fruit color using reference-based (GATK, SVGAP, and Panpop) and reference-free (PGGB) methods. **d**, **e**, **f**, Genome-wide association analyses based on SNPs, InDels, and SVs for bacterial wilt resistance using reference-based (GATK, SVGAP, and Panpop) and reference-free (PGGB) methods, respectively. *P* values were calculated using a two-sided Wald test based on a linear mixed model.

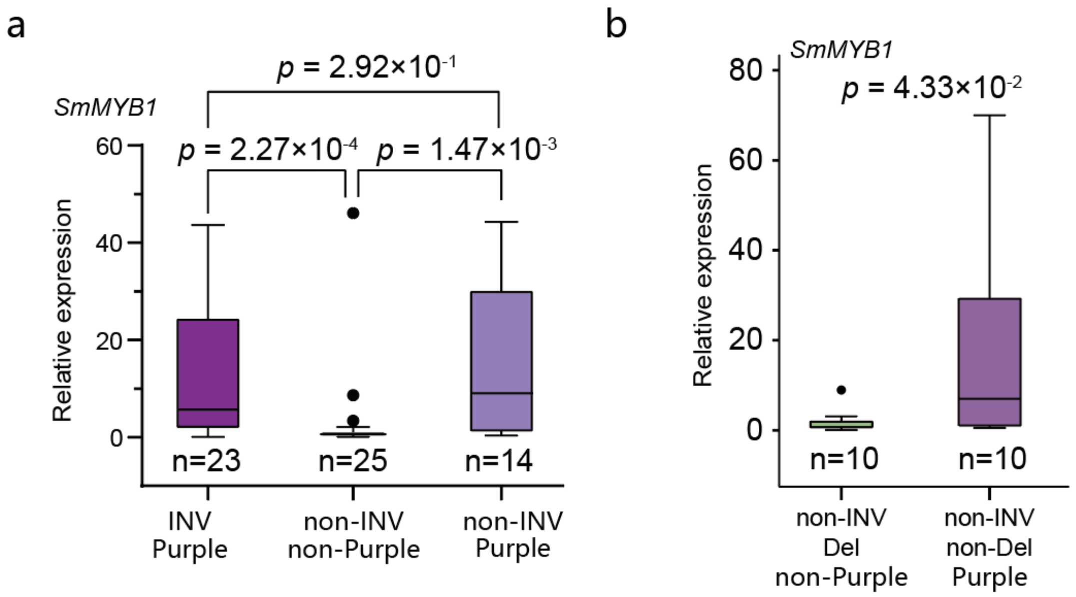

Supplementary Fig. 15. Comparison of expression levels of *SmMYB1* among eggplant accessions with different genotypes at the 12.4-Mb inversion and the InDel loci by qRT-PCR. a, Pairwise comparison of the expression of *SmMYB1* between different eggplant groups. 1) Accessions with purple fruit and the inversion *vs.* accessions with purple fruit without the inversion; 2) Accessions with purple fruit and the inversion *vs.* accessions with non-purple fruit without the inversion; 3) Accessions with purple fruit without the inversion *vs.* accessions with non-purple fruit without the inversion. **b**, Comparison of expressions of *SmMYB1* between accessions with deletion at the 6-bp InDel and non-purple and accessions with non-deletion at the 6-bp InDel and purple. In boxplots (**a, b**), the lower and upper edges of the box represent the first and third quartiles, respectively, and the central line indicates the median. The whiskers extend to the smallest and largest values within 1.5 × IQR. The p values above the bars indicate significantly different values ($p < 0.05$) calculated using unpaired t-test (two sided). Source data are provided as a Source Data file.

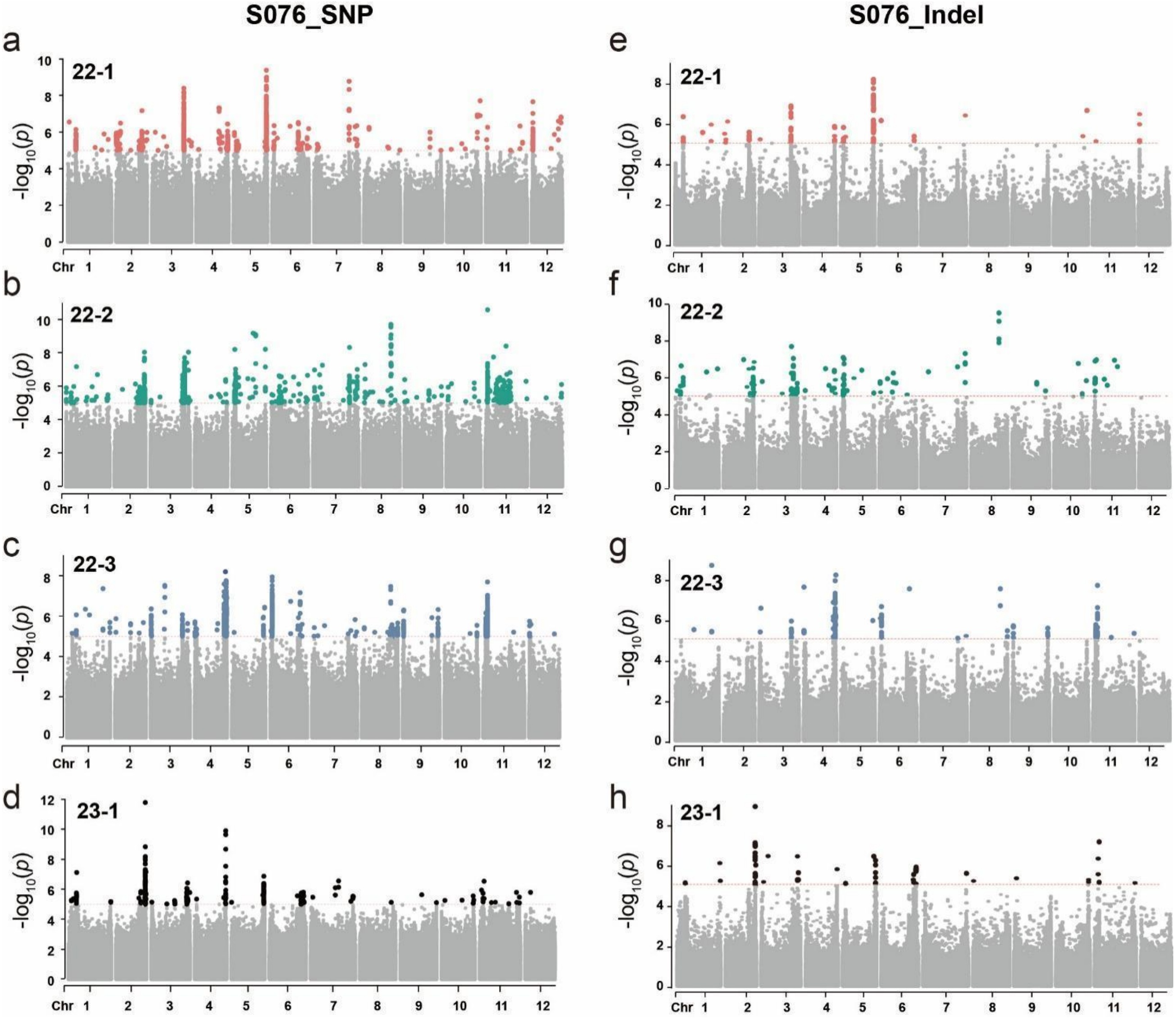

Supplementary Fig. 16. GWAS analysis for bacterial wilt resistance in eggplant using S076 as the reference. a, SNP-GWAS for the first batch (22-1). **b**, SNP-GWAS for the second batch (22-2). **c**, SNP-GWAS for the third batch (22-3). **d**, SNP-GWAS for the fourth batch (23-1). **e,** InDel-GWAS for the first batch (22-1). **f,** InDel-GWAS for the second batch (22-2). **g,** InDel-GWAS for the third batch (22-3). **h**, InDel-GWAS for the fourth batch (23-1). *P* values were calculated using a two-sided Wald test based on a linear mixed model.

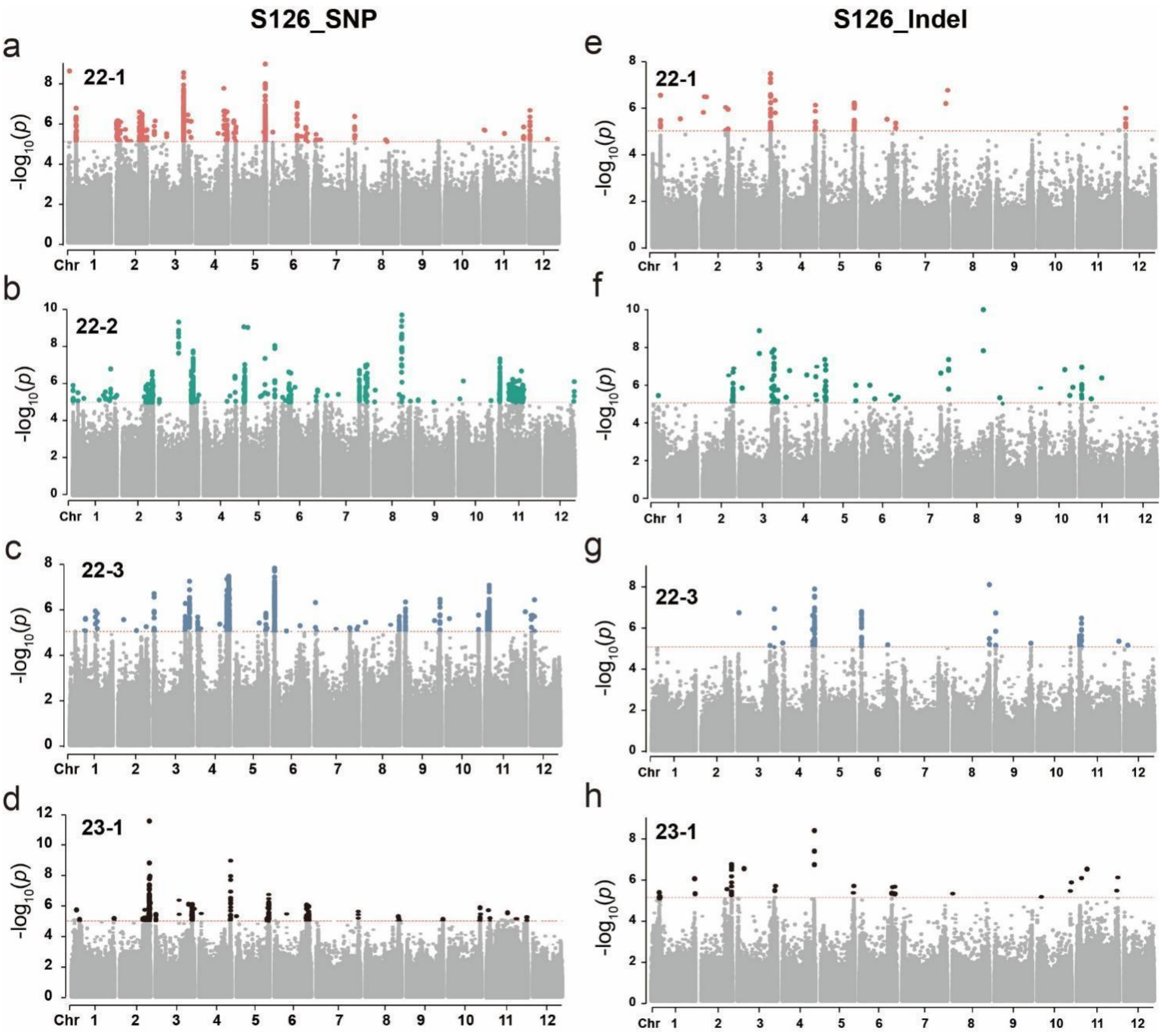

Supplementary Fig. 17. GWAS analysis for bacterial wilt resistance in eggplant using S126 as the reference. **a**, SNP-GWAS for the first batch (22-1). **b**, SNP-GWAS for the second batch (22-2). **c**, SNP-GWAS for the third batch (22-3). **d**, SNP-GWAS for the fourth batch (23-1). **e**, InDel-GWAS for the first batch (22-1). **f**, InDel-GWAS for the second batch (22-2). **g**, InDel-GWAS for the third batch (22-3). **h**, InDel-GWAS for the fourth batch (23-1). *P* values were calculated using a two-sided Wald test based on a linear mixed model.

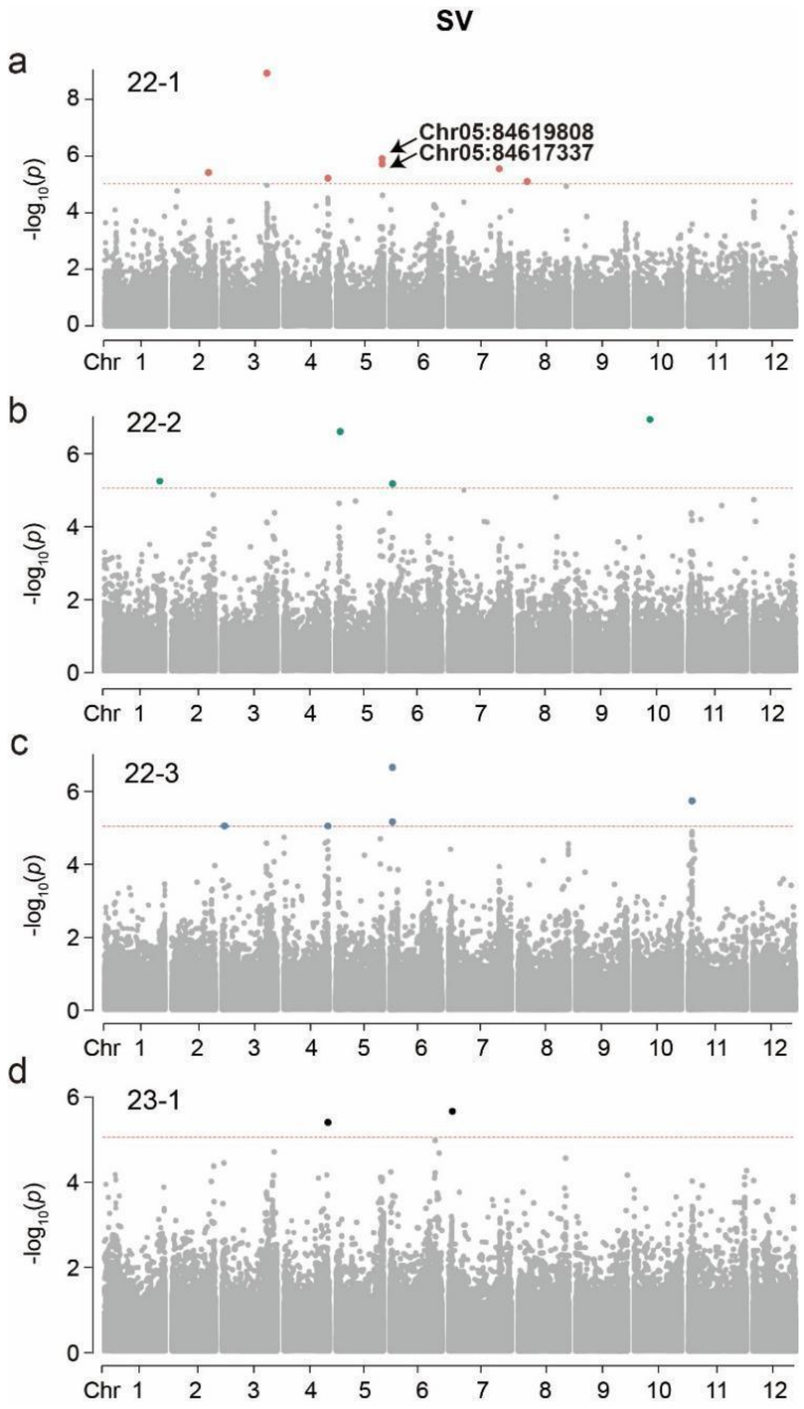

Supplementary Fig. 18. GWAS analysis for bacterial wilt resistance in eggplant using a unique SV dataset. a, GWAS for the first batch (22-1). The two SV signals on Chr05 are highlighted with arrows. **b**, GWAS for the second batch (22-2). **c**, GWAS for the third batch (22-3). **d**, GWAS for the fourth batch (23-1). *P* values were calculated using a two-sided Wald test based on a linear mixed model.

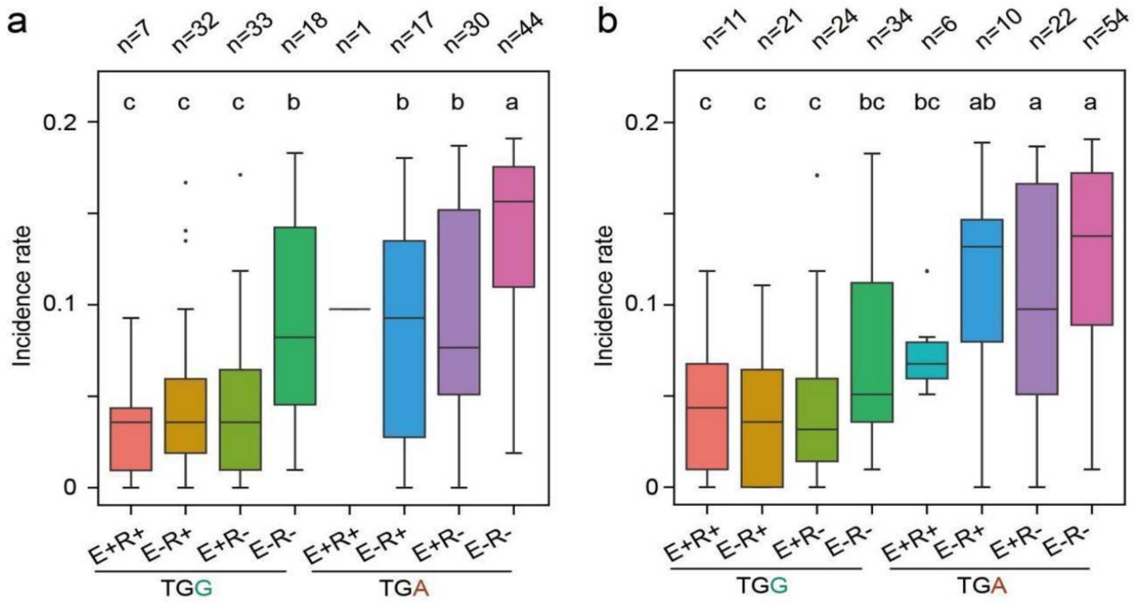

Supplementary Fig. 19. Comparison of bacterial wilt incidence rates for 182 accessions with different genotype combinations defined by two approaches for *SmCYP82D47*, *SmEPS1* and *SmRoq1*. **a**, Comparison based on *SmCYP82D47*, SVe, and SVr; **b**, Comparison based on *SmCYP82D47* and normalized mean coverages of *SmEPS1* and *SmRoq1*. Normalized average incidence rates are plotted. In boxplots (**a, b**), the lower and upper edges of the box represent the first and third quartiles, respectively, and the central line indicates the median. The whiskers extend to the smallest and largest values within 1.5 × IQR. Different letters (a, b, c, etc.) above the bars indicate significantly different values ($p < 0.05$) calculated using the Wilcoxon rank-sum test (two sided) for **a** and **b**.

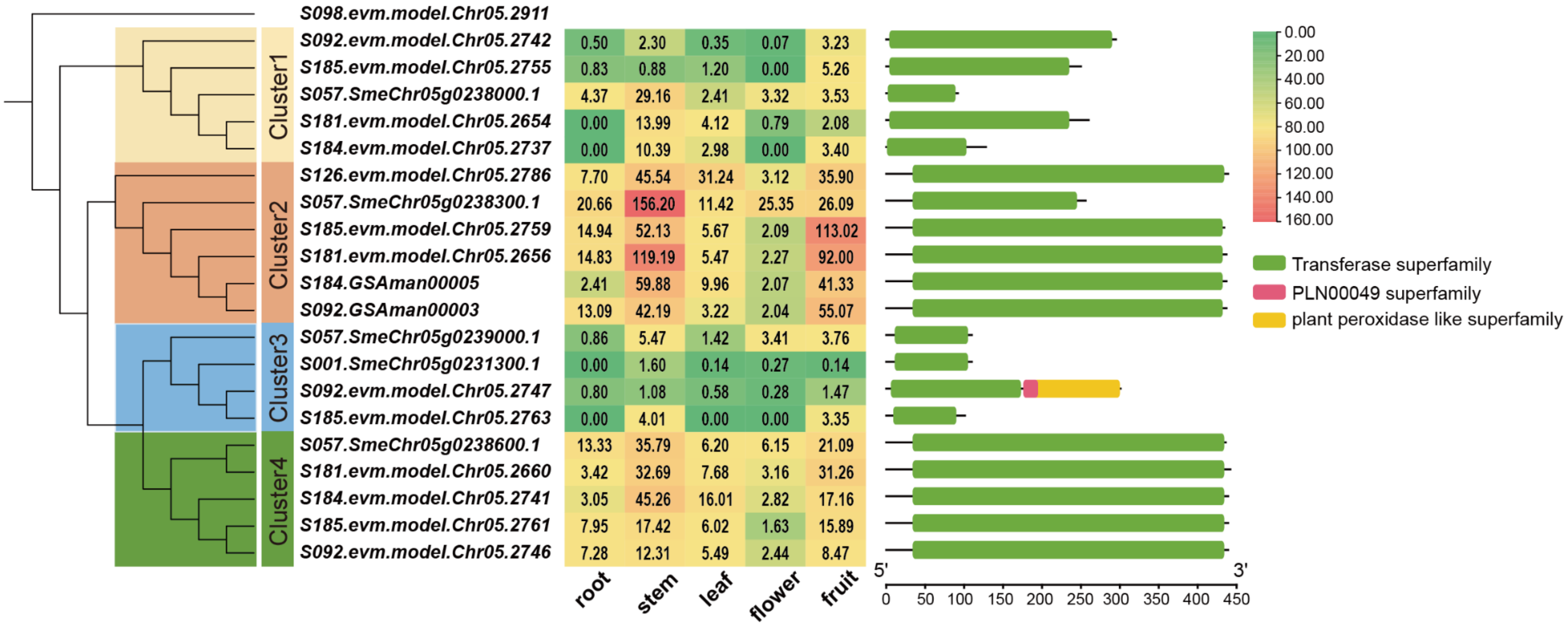

Supplementary Fig. 20. Comparison of gene expressions and protein lengths for *SmEPS1* homologs. The EPS1 homolog from S098 was used as the outgroup. Values in the heatmap are TPM expression values. The domains are shown in different colors. Source data are provided as a Source Data file.

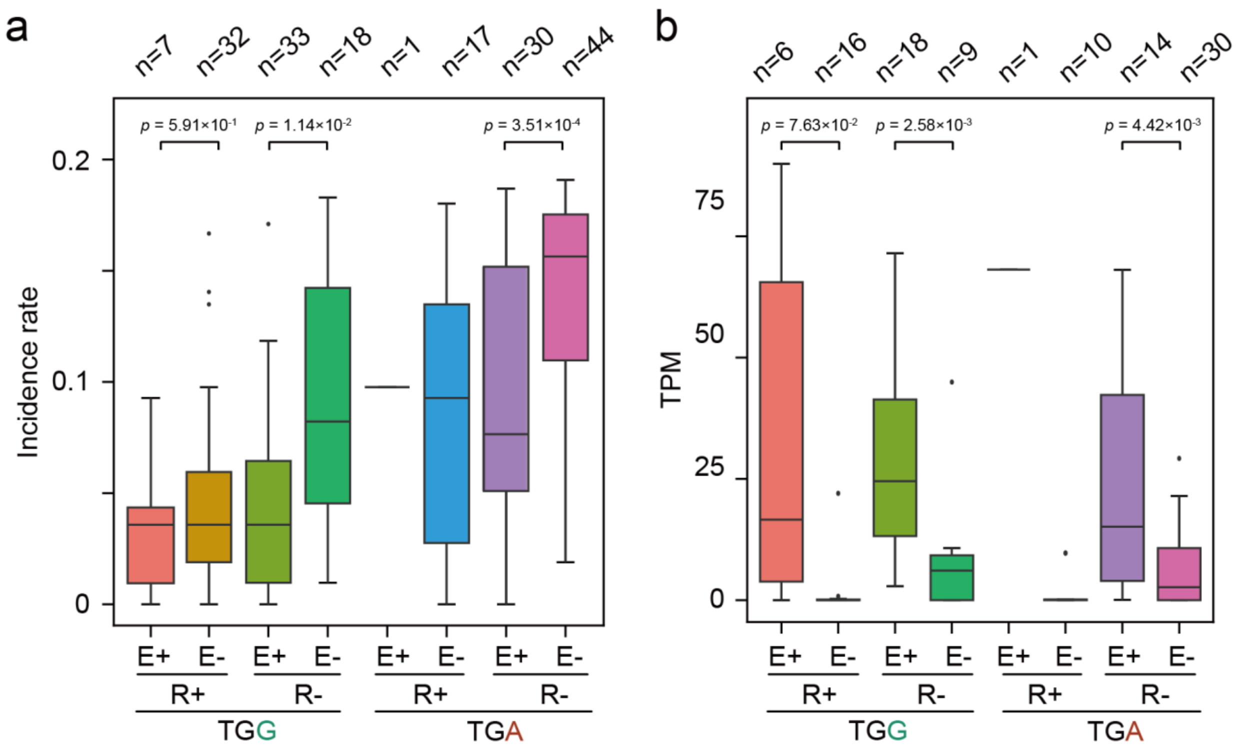

Supplementary Fig. 21. Comparison of *SmEPS1* gene expressions across samples with different genotype combinations of the three gene classes and with different resistance levels. a, The disease incidence rate for different genotype combinations of the three gene classes. **b**, the TPM expression values of *SmEPS1* for different genotype combinations of the three gene classes. In boxplots (**a, b**), the lower and upper edges of the box represent the first and third quartiles, respectively, and the central line indicates the median. The whiskers extend to the smallest and largest values within 1.5 × IQR. The p values indicate significantly different values ($p < 0.05$) calculated using Wilcoxon rank-sum test (two sided) for **a** and **b**.

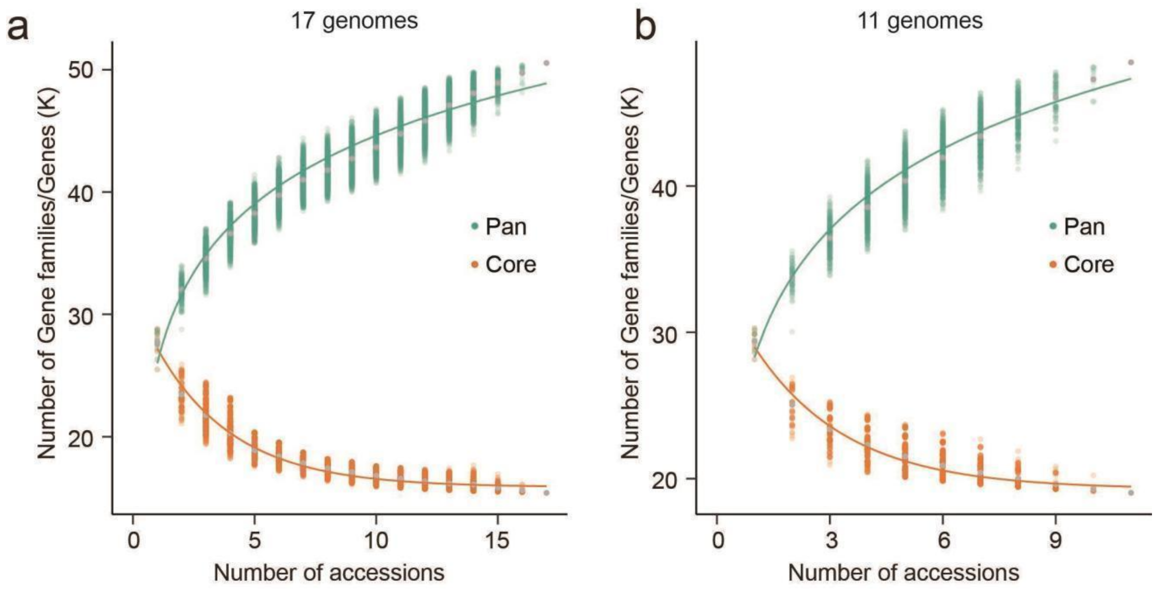

Supplementary Fig. 22. Pan and core gene families/genes considering all genes. a, Number of pan and core gene families/genes considering all 17 genomes; b, Number of pan and core gene families/genes considering only de novo sequenced genomes.

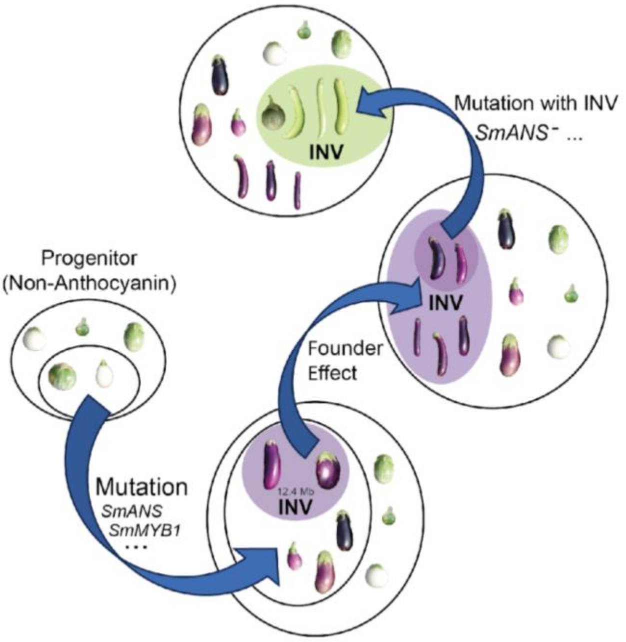

Supplementary Fig. 23. A proposed model illustrating the origin and evolutionary trajectory of a 12.4-Mb inversion and its association with fruit color during the domestication and breeding history of eggplant. First, early cultivated eggplants were likely domesticated from wild progenitors lacking a complete anthocyanin biosynthesis pathway, resulting in non-anthocyanic fruits with white or green peels. Second, semi-cultivated eggplants likely continued to hybridize with wild individuals, or accumulated mutations in the related genes of the anthocyanin biosynthesis pathway—such as *SmANS* and *SmMYB1*—thereby reconstituting a complete pathway and leading to the emergence of purple-peeled eggplants. Third, at a certain point, a 12.4-Mb chromosomal inversion occurred in purple-peeled individuals, with breakpoints near the *SmMYB1* gene, a key regulator of eggplant peel color, and possibly other genetic factors influencing purple fruit formation. Although this large inversion does not directly affect the expression of *SmMYB1* or other genetic features, it is closely linked to a combination of genetic factors controlling the development of purple fruit and therefore tightly linked with the purple color. Fourth, purple fruits are desirable breeding targets in certain regions, such as in China. During the eggplant breeding process, a limited number of founder plants with purple fruits and carrying the 12.4-Mb inversion may have increased its frequency in cultivated varieties and spread to other regions through hybridization and recombination. Fifth, the association between the inversion and the genetic factors controlling fruit color formation can be disrupted due to breeding practices, hybridization, and natural variations. For example, a rare mutation leading to a premature stop codon in the *SmANS* gene has been identified. This mutation, present in less than 5% of the studied population, results in a small proportion of purple-peeled eggplants with the inversion exhibiting non-purple peels.

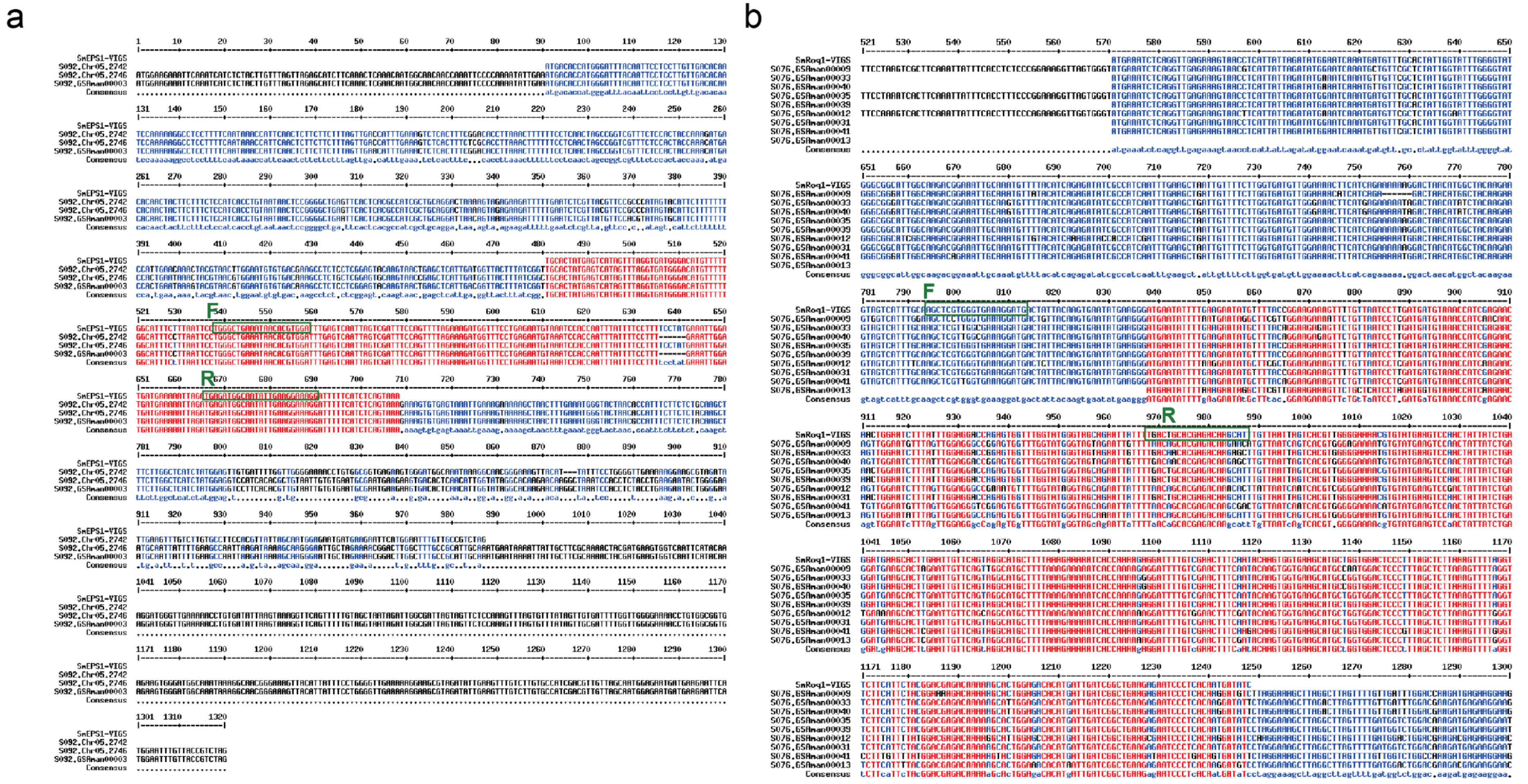

Supplementary Fig. 24. Comparison of VIGS fragments with homologs of *SmEPS1* and *SmRoq1*. a, Sequence alignments with *SmEPS1* homologs, and the conserved region was highlighted in red. **b**, Sequence alignments with *SmRoq1* homologs, and the conserved region was highlighted in red. F denotes the primer-binding sites of the forward primers, and R denotes those of the reverse primers.

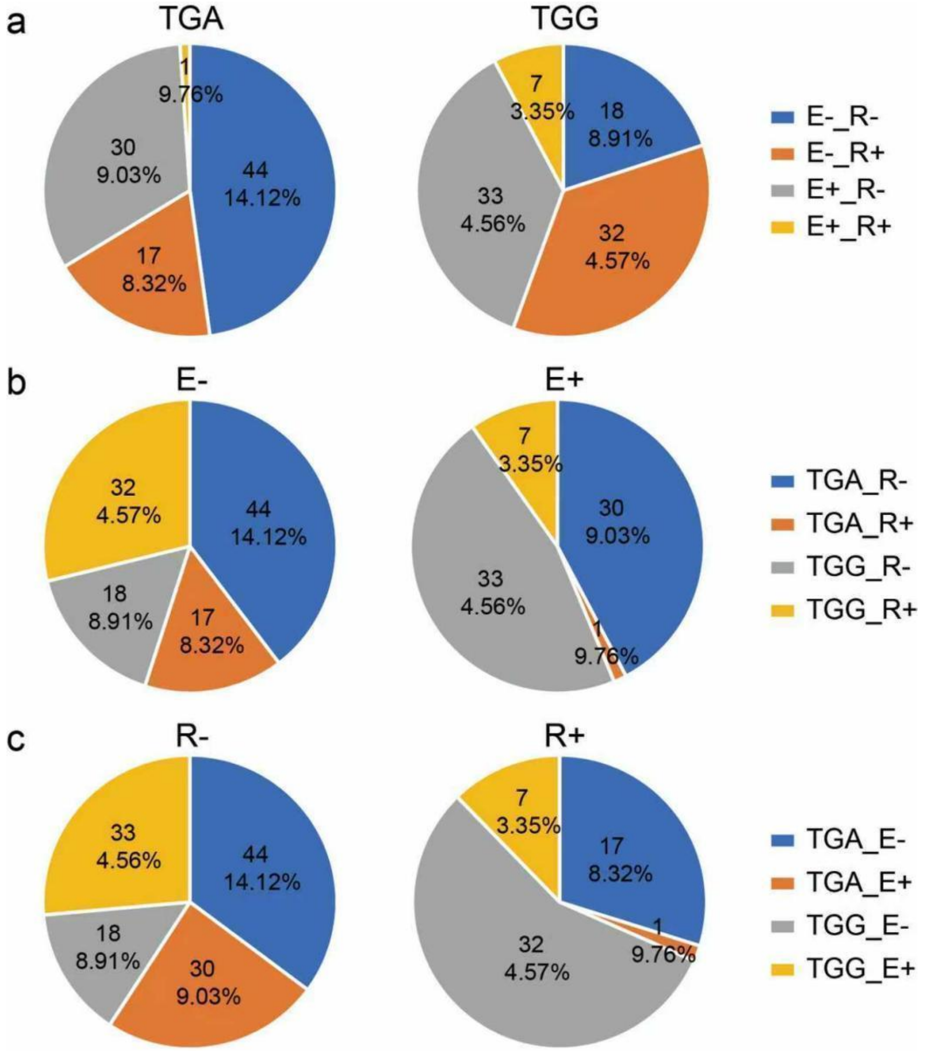

Supplementary Fig. 25. Distribution of *SmCYP82D47*, *SmEPS1* (*E*), and *SmRoq1* (*R*) genotypes and their association with bacterial wilt resistance in eggplant. a, Pie charts showing the proportion of E+R+, E-R+, E+R-, and E-R- genotypes in TGG and TGA genetic backgrounds. **b**, Pie charts showing the proportion of TGA_R+, TGA_R-, TGG_R+, and TGG_R- genotypes in E- and E+ genetic backgrounds. **c**, Pie charts showing the proportion of TGA_E+, TGA_E-, TGG_E+, and TGG_E- genotypes in R+ and R- genetic backgrounds. The number of accessions and the average susceptibility (%) to *Ralstonia solanacearum* are indicated for each genotype class in pie charts.

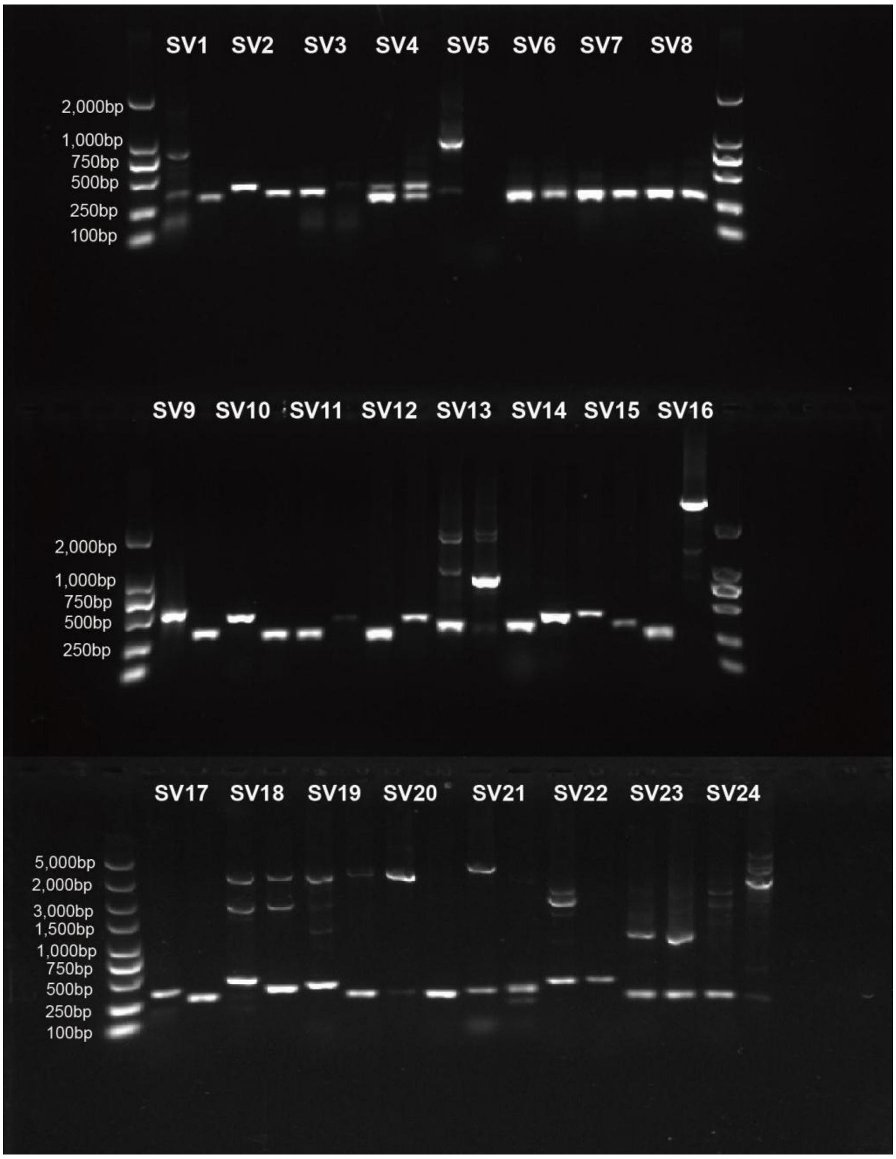

Supplementary Fig. 26. PCR amplification and validation of structural variants (SVs). A total of 24 SVs were randomly selected for validation. DNA samples from S076 and S054 were used as templates. Source data are provided as a Source Data file.

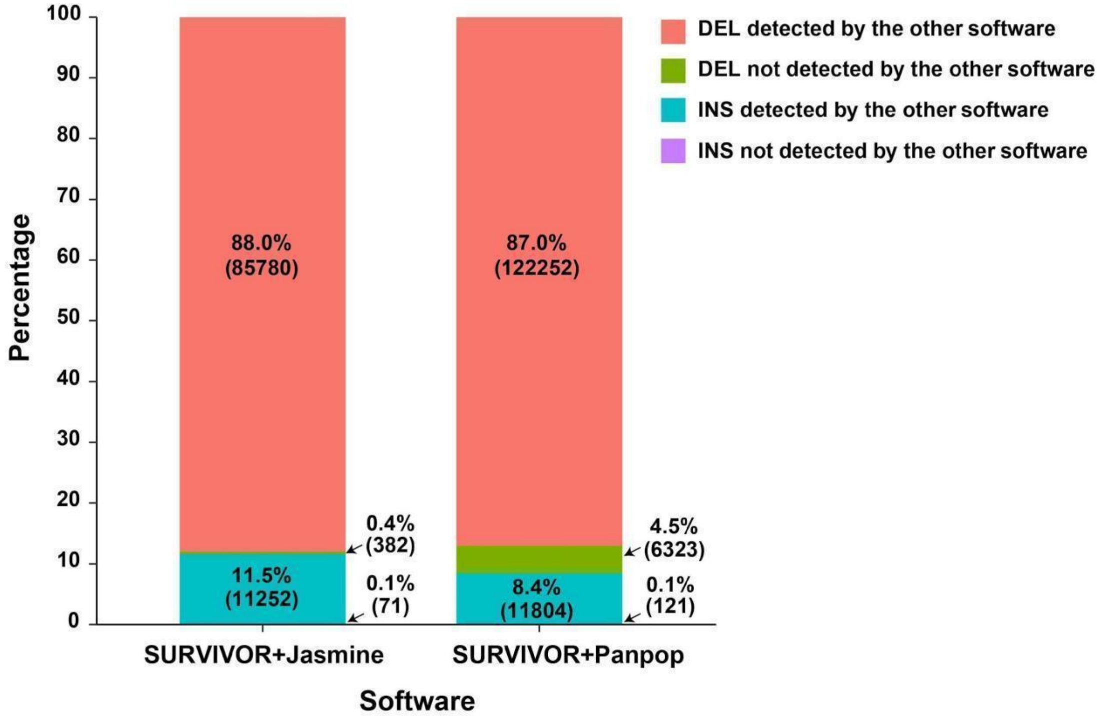

Supplementary Fig. 27. Comparison of two SV merging methods: SURVIVOR combined with Jasmine, and SURVIVOR combined with PanPop. The results demonstrate high consistency between the two approaches: 99.5% of SVs detected by SURVIVOR + Jasmine are also identified by SURVIVOR + PanPop, while 95.4% of SVs detected by SURVIVOR + PanPop are also found by SURVIVOR + Jasmine.
