# Supplementary material for: Sub-pangenome analysis reveals structural variants associated with fruit color and bacterial wilt resistance in eggplant
Source: Nat Commun. 2026 Feb 23;17:3075. doi: 10.1038/s41467-026-69764-8 (PMC13039718; doi:10.1038/s41467-026-69764-8)
Supplement: Supplementary file 3 — Description of Additional Supplementary Files [file 41467_2026_69764_MOESM3_ESM.pdf]

Description of Additional Supplementary Files

File Name: Supplementary Data 1
Description: Summary of 226 eggplant germplasms for whole genome re-sequencing.

File Name: Supplementary Data 2
Description: Genome assembly and annotation statistics of the 11 de novo assembled and published genomes.

File Name: Supplementary Data 3
Description: Gene ontology (GO) enrichment in defense-related terms for either 17 or 11 eggplant genomes.

File Name: Supplementary Data 4
Description: The number of structural variants identified between S076 and each of the other 15 eggplant accessions using the pairwise whole-genome alignment method.

File Name: Supplementary Data 5
Description: The coordinates (according to S076) of large inversions (approximately 5 Mb or larger) that are segregating within the eggplant population identified in our sample.

File Name: Supplementary Data 6
Description: The number of the derived variants inferred using S098 as the outgroup.

File Name: Supplementary Data 7
Description: SV-affected genes that are significantly differentially expressed in fruit.

File Name: Supplementary Data 8
Description: Comparison of the variation total numbers used ref-based (GATK, SVGAP) and ref-free (PGGB) methods.

File Name: Supplementary Data 9
Description: Candidate genes and variations associated with fruit color identified by SNP-GWAS, InDel-GWAS, and SV-GWAS.

File Name: Supplementary Data 10
Description: Comparison of the variation numbers associated with fruit color and bacterial wilt resistance used GATK, SVGAP and PGGB methods.

File Name: Supplementary Data 11
Description: Genotypes of Inversion and InDel associated with fruit color.

File Name: Supplementary Data 12
Description: The bacterial wilt incidence rate in 197 eggplant accessions in 2022 and 2023.

File Name: Supplementary Data 13
Description: Variants and candidate genes associated with bacterial wilt resistance.

File Name: Supplementary Data 14
Description: Candidate EPS1 and Roq1-like homologs in 11 genomes.

File Name: Supplementary Data 15
Description: PGGB derived SNPs, InDels, and SVs associated with four candidate genes for fruit color and bacterial wilt resistance.

File Name: Supplementary Data 16
Description: Genes previously reported associated with bacterial wilt resistance in eggplant.

File Name: Supplementary Data 17
Description: Homology levels between VIGS target fragments and their corresponding genes.

File Name: Supplementary Data 18
Description: Primers used for cloning, qRT-PCR, VIGS, and overexpression.
