# Supplementary material for: Sub-pangenome analysis reveals structural variants associated with fruit color and bacterial wilt resistance in eggplant
Source: Nat Commun. 2026 Feb 23;17:3075. doi: 10.1038/s41467-026-69764-8 (PMC13039718; doi:10.1038/s41467-026-69764-8)
Supplement: Supplementary file 22 — Reporting Summary [file 41467_2026_69764_MOESM22_ESM.pdf]

Corresponding author(s): Qian You, Yi Liao

Last updated by author(s): Feb 3, 2026

Reporting Summary

Nature Portfolio wishes to improve the reproducibility of the work that we publish. This form provides structure for consistency and transparency in reporting. For further information on Nature Portfolio policies, see our Editorial Policies and the Editorial Policy Checklist.

Statistics

For all statistical analyses, confirm that the following items are present in the figure legend, table legend, main text, or Methods section.

n/a	Confirmed	
☐	☒	The exact sample size (*n*) for each experimental group/condition, given as a discrete number and unit of measurement
☐	☒	A statement on whether measurements were taken from distinct samples or whether the same sample was measured repeatedly
☐	☒	The statistical test(s) used AND whether they are one- or two-sided<br>*Only common tests should be described solely by name; describe more complex techniques in the Methods section.*
☒	☐	A description of all covariates tested
☐	☒	A description of any assumptions or corrections, such as tests of normality and adjustment for multiple comparisons
☐	☒	A full description of the statistical parameters including central tendency (e.g. means) or other basic estimates (e.g. regression coefficient) AND variation (e.g. standard deviation) or associated estimates of uncertainty (e.g. confidence intervals)
☐	☒	For null hypothesis testing, the test statistic (e.g. *F*, *t*, *r*) with confidence intervals, effect sizes, degrees of freedom and *P* value noted<br>*Give P values as exact values whenever suitable.*
☒	☐	For Bayesian analysis, information on the choice of priors and Markov chain Monte Carlo settings
☒	☐	For hierarchical and complex designs, identification of the appropriate level for tests and full reporting of outcomes
☐	☒	Estimates of effect sizes (e.g. Cohen's *d*, Pearson's *r*), indicating how they were calculated

Our web collection on statistics for biologists contains articles on many of the points above.

Software and code

Policy information about availability of computer code

Data collection	A detailed description of the sofwares/packages/tools used for data collection are described in the Methods section.
Data analysis	A detailed description of the software/packages/tools used for data analysis is provided in the Methods section. Here is a summary: Hifiasm (v0.16.1), ALLHiC (v0.9.8), Juicebox (Durand et al. 2016), Mummer (v4.0.0rc1), BUSCO (v.5.2.2), BWA (v2.2.1), TRF (v4.09), RepeatMasker (v4.1.0), LTR_FINDER (v1.06), RepeatScout (v1.0.5), RepeatModeler (v2.0.1), GeneWise (v2.4.1), Augustus (v3.2.3), Geneid (v1.4), Genescan (v1.0), GlimmerHMM (v3.04), SNAP (2013-11-29), Trinity (v2.1.1), HISAT (v.2.0.4), StringTie2 (v.1.3.3), EvidenceModeler (EVM, v1.1.1), tRNAscan-SE (v1.4), Rfam (v14.1), InterProScan (v.5.31), Trimmomatic (v0.39), BWA (v2.2.1), GATK (v4.2.6.1), Plink (v0.98.3), iTOL (v6), ADMIXTURE (v1.30), TBbtools (v1.123), VCFtools (v0.1.16), PopLDdecay (v3.41), SvABA (v1.1.0), DELLY (v1.1.6), Manta (v1.6.0), SURVIVOR (v1.0.7), OrthoFinder (v.2.5.4), GEMMA (v.0.98.1), CMplot (v4.4.1), qqman (v0.1.9), SVGAP, Panpon, and PGGB (v0.7.4).<br>Codes used in this study are available at https://github.com/yiliao1022/eggplantpangenome (https://doi.org/10.5281/zenodo.18467477) .

For manuscripts utilizing custom algorithms or software that are central to the research but not yet described in published literature, software must be made available to editors and reviewers. We strongly encourage code deposition in a community repository (e.g. GitHub). See the Nature Portfolio guidelines for submitting code & software for further information.

Data

Policy information about availability of data

All manuscripts must include a data availability statement. This statement should provide the following information, where applicable:

- Accession codes, unique identifiers, or web links for publicly available datasets
- A description of any restrictions on data availability
- For clinical datasets or third party data, please ensure that the statement adheres to our policy

All the raw sequencing data, genome assemblies and annotations have been submitted to China National GeneBank (CNGB) database under Project accession number CNP0006177. The variant (VCF) and pangenome graph files have been deposited in the Zenodo database (https://doi.org/10.5281/zenodo.18425195). Source Data is provided with this paper.

Research involving human participants, their data, or biological material

Policy information about studies with human participants or human data. See also policy information about sex, gender (identity/presentation), and sexual orientation and race, ethnicity and racism.

Reporting on sex and gender	N/A
Reporting on race, ethnicity, or other socially relevant groupings	N/A
Population characteristics	N/A
Recruitment	N/A
Ethics oversight	N/A

Note that full information on the approval of the study protocol must also be provided in the manuscript.

Field-specific reporting

Please select the one below that is the best fit for your research. If you are not sure, read the appropriate sections before making your selection.

- [x] Life sciences
- [ ] Behavioural & social sciences
- [ ] Ecological, evolutionary & environmental sciences

For a reference copy of the document with all sections, see nature.com/documents/nr-reporting-summary-flat.pdf

Life sciences study design

All studies must disclose on these points even when the disclosure is negative.

Sample size	A total of 226 eggplant germplasm accessions were collected from 20 provinces in China, Southeast Asia, South America, and Europe, among other regions. This collection includes: 219 accessions of Solanum melongena (the cultivated eggplant), two accessions of its closest wild relatives: S. insanum and S. incanum, four accessions of scarlet eggplant (S. aethiopicum), one accession of wild eggplant (S. violaceum). Out of these, 198 accessions were newly resequenced. Additionally, we performed de novo sequencing on 11 accessions, including S076, along with six previously published accessions. This set, totaling 17 accessions, represents the major clusters of a phylogenetic tree derived from the sequencing of 230 eggplant accessions, which were analyzed for the pangenome study.
Data exclusions	Only one wild eggplant species (S. violaceum) was excluded for GWAS among the 198 new resequenced accessions.
Replication	We have performed all the experiments in replications and results are reproducible.
Randomization	The plants were randomly allocated in the field or in a disease nursery.
Blinding	Blinded data were not relevant to our study.

Reporting for specific materials, systems and methods

We require information from authors about some types of materials, experimental systems and methods used in many studies. Here, indicate whether each material, system or method listed is relevant to your study. If you are not sure if a list item applies to your research, read the appropriate section before selecting a response.

Materials & experimental systems

n/a	Involved in the study	
☒	☐	Antibodies
☒	☐	Eukaryotic cell lines
☒	☐	Palaeontology and archaeology
☒	☐	Animals and other organisms
☒	☐	Clinical data
☒	☐	Dual use research of concern
☐	☒	Plants

Methods

n/a	Involved in the study	
☒	☐	ChIP-seq
☒	☐	Flow cytometry
☒	☐	MRI-based neuroimaging

Dual use research of concern

Policy information about dual use research of concern

Hazards

Could the accidental, deliberate or reckless misuse of agents or technologies generated in the work, or the application of information presented in the manuscript, pose a threat to:

No	Yes	
☒	☐	Public health
☒	☐	National security
☒	☐	Crops and/or livestock
☒	☐	Ecosystems
☒	☐	Any other significant area

Experiments of concern

Does the work involve any of these experiments of concern:

No	Yes	
☒	☐	Demonstrate how to render a vaccine ineffective
☒	☐	Confer resistance to therapeutically useful antibiotics or antiviral agents
☒	☐	Enhance the virulence of a pathogen or render a nonpathogen virulent
☒	☐	Increase transmissibility of a pathogen
☒	☐	Alter the host range of a pathogen
☒	☐	Enable evasion of diagnostic/detection modalities
☒	☐	Enable the weaponization of a biological agent or toxin
☒	☐	Any other potentially harmful combination of experiments and agents

Plants

Seed stocks	Information regarding the seed stocks related to the eggplant genotypes used in this study is reported in Supplementary Data 1.
Novel plant genotypes	No novel plant genotypes have been developed in this study.
Authentication	N/A
